# Pseudo-Pseudo Meigs’ Syndrome in a 40-Year-Old Woman Without a Prior Systemic Lupus Erythematosus Diagnosis

**DOI:** 10.7759/cureus.74020

**Published:** 2024-11-19

**Authors:** Salome Tsertsvadze, Dimitri Daraselia, Rusudan Tskitishvili, Ana Eradze, Nino Kantaria

**Affiliations:** 1 Internal Medicine, Tbilisi State Medical University, Tbilisi, GEO; 2 Internal Medicine, New Hospitals, Tbilisi, GEO

**Keywords:** elevated ca-125, pseudo-pseudo meigs’ syndrome, serositis, systemic lupus erythematosus, tjalma syndrome

## Abstract

Pseudo-pseudo Meigs’ syndrome (PPMS), also known as Tjalma syndrome, is an exceptionally rare condition marked by pleural effusion, ascites, and elevated CA-125 levels, usually in the context of systemic lupus erythematosus (SLE) without any associated ovarian tumors. We report the case of a 40-year-old woman who presented with a four-month history of fatigue, nausea, weight loss, abdominal pain, and pleural effusion. Initial diagnostic workup revealed ascites, elevated CA-125 levels, and pleural effusions, with no evidence of malignancy. Notably, the patient had no prior history of SLE. Comprehensive clinical, biological, and immunological evaluation eventually confirmed SLE, leading to a diagnosis of PPMS. The patient was treated with methylprednisolone and Plaquenil, with careful monitoring for potential complications. This case highlights the diagnostic challenges posed by PPMS, particularly in the absence of a prior SLE diagnosis. The unusual initial presentation underscores the importance of considering PPMS in differential diagnoses to avoid misdiagnosis and unnecessary invasive procedures. Early recognition and appropriate management are essential for optimizing patient outcomes in this rare and complex syndrome.

## Introduction

Although the combination of ascites, pleural effusion, and elevated CA-125 levels is usually associated with a gynecological malignancy, it may also occur with Pseudo-pseudo Meigs’ syndrome (PPMS), which has no association with benign or malignant pelvic tumors. Meigs’ syndrome stands in contrast to pseudo-Meigs, which presents with ascites and pleural effusion in association with benign tumors of the ovary (other than fibromas) and malignant tumors. PPMS is a rare condition characterized by a triad of ascites, pleural effusion, and elevated CA-125 in a patient with systemic lupus erythematosus (SLE) [1]. SLE is a chronic inflammatory disease characterized by gradual-onset ascites, which is recognized sometime after diagnosis. In contrast, massive ascites is an uncommon initial presentation of SLE and has rarely been reported in the literature [2,3]. We have little clinical information on this condition, with only 15 cases reported so far. Such an unusual initial manifestation of SLE can be misdiagnosed if there is a lack of classic symptoms of the disease, leading to inappropriate treatment and unnecessary invasive investigations or surgery. This case informs fellow physicians about the symptoms of Tjalma syndrome and encourages them to consider it as a differential diagnosis

## Case presentation

A 40-year-old woman presented to the clinic with a four-month history of fatigue, nausea, recurrent emesis, ascites, abdominal pain, frequent watery diarrhea with mucus, unintentional urination and defecation, and weight loss of 15 kg. She had no history of fever, skin rashes, or inflammatory joint pain. The patient’s medical history was significant for Raynaud’s phenomenon for an extended period. Additionally, her family history included leukemia in her mother, who died at the age of 40. Physical examination revealed a distended and soft abdomen with slight umbilical tenderness, shifting dullness, and a positive fluid thrill. No organomegaly or lymphadenopathy was noted.

The patient had been previously admitted to our hospital with frequent diarrhea and abdominal pain. At that time, investigations revealed positive Clostridium difficile toxins, and appropriate treatment was initiated. After stabilizing the patient’s clinical symptoms and lab results, she was discharged.

**Figure 1:**
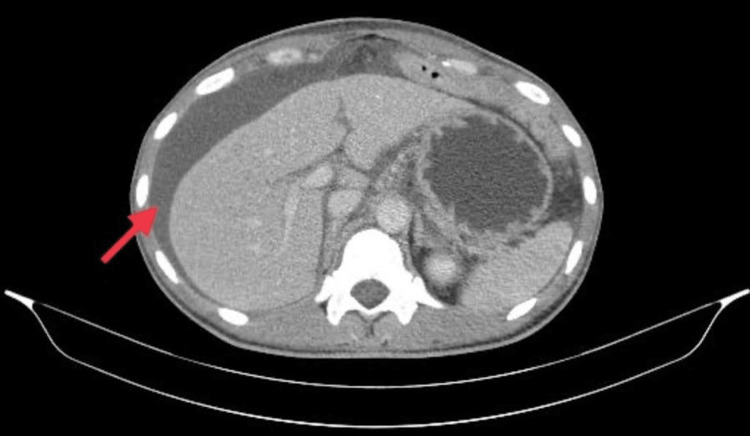
CT scan of the abdomen demonstrating peritoneal fluid

The patient’s lab tests showed hypokalemia of 2.2 mmol/L, albumin levels of 26.8 g/L, and creatinine of 160 μmol/L. Liver function tests were normal, and Clostridium difficile toxins were negative. Clinical findings and laboratory results showed no evidence of active infection. CT scan of the abdomen demonstrated ascites (Figure 1). Abdominal ultrasound revealed free fluid, approximately 4000 mL. Diagnostic abdominal paracentesis showed a serum ascites albumin gradient of <1 with no malignant cells. Fluid analysis revealed a white cell count of 237/μL with 88.2% mononuclear cells, suggesting exudative ascites.

**Figure 2:**
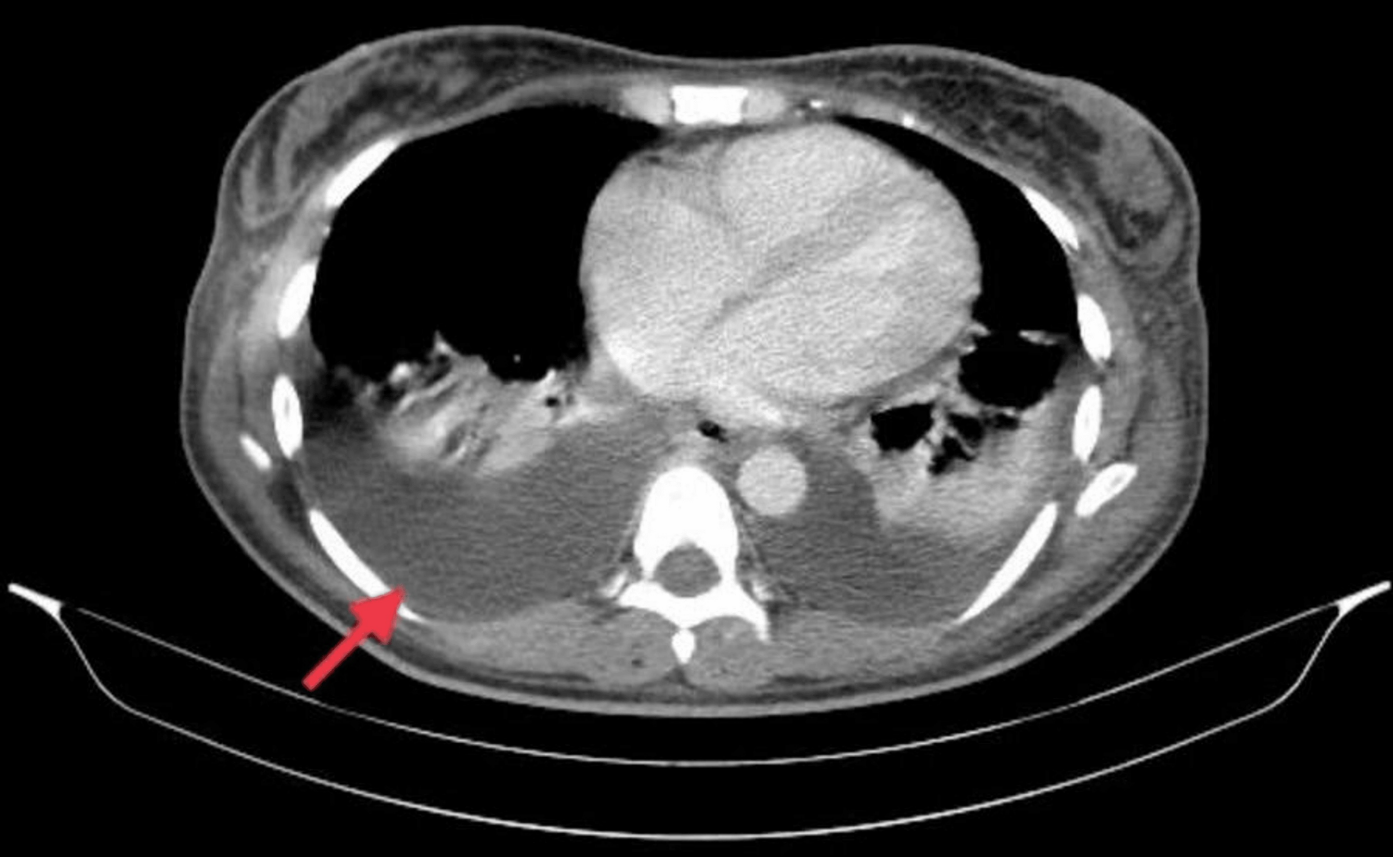
Chest CT scan revealed bilateral pleural effusions

A chest CT scan revealed bilateral pleural effusions with separations of 7.4 cm on the right and 5.4 cm on the left sides (Figure 2). Left-side thoracentesis yielded 2000 mL of fluid, and lab tests of the punctate revealed exudate with excessive lymphocytes. Suspecting malignancy (Meigs syndrome or Pseudo-Meigs syndrome) human immunodeficiency virus was excluded through specific testing. Oncomarker tests were conducted: CEA 0.693 ng/mL, CA15.3 16.47 U/mL (normal range <25 U/mL), CA19.9 8.04 U/mL (normal range <27 U/mL), and CA125 553.4 U/L (positive, normal range <35 U/L). An abdominal MRI showed entire thickening of the colon and ileum, moderate-to-gross ascites, and normal uterus and ovaries. Previously, the patient’s colonoscopy showed erosive sigmoiditis, and the biopsy result revealed inflammatory changes without malignancy. Cytology of pleural and ascitic fluid showed no atypical cells, and ADA was negative, effectively excluding tuberculosis.

Based on her symptoms and findings, autoimmune markers were investigated, revealing a positive antinuclear antibody (ANA) of 7.2 (<1.0) and anti-double stranded DNA (dsDNA) of 47.5 IU/mL (normal range <25 IU/mL) (Table 1). ANA Hep-2 showed nuclear coarse speckles in high titers (1:1280), a cytoplasmic fluorescence pattern (1:640), low C3 (0.36), and low C4 (0.06). Anti-cardiolipin antibodies (IgG and IgM) were negative. According to the new ACR/EULAR classification criteria, SLE was diagnosed, with the patient scoring a total of 11 points. Additionally, based on the patient’s findings of pleural effusion, ascites, elevated CA-125 with no associated benign or malignant ovarian tumor, and newly diagnosed SLE, Tjalma syndrome (or PPMS) was diagnosed.

**Table 1:** Relevant laboratory findings for a 40-year-old patient with PPMS PPMS: pseudo-pseudo Meigs' syndrome; ANA: antinuclear antibody; dsDNA: double stranded DNA; SLE: systemic lupus erythematosus

Test	Value	Normal range	Relevance to SLE / PPMS
CA-125	553.4 U/L	<35 U/L	Markedly elevated; indicative of PPMS when no ovarian tumor is present. Elevated SLE in some cases
White cell count (ascitic fluid)	237/μL	<250/μL (exudative)	Elevated; supports exudative ascites, relevant for PPMS
Mononuclear cells (ascitic fluid)	88.20%	-	A high percentage indicates the exudative nature of ascites, relevant for PPMS
ANA	7.2	<1.0	Highly elevated; suggestive of SLE
Anti-dsDNA	47.5 IU/mL	<25 IU/mL	Elevated; specific for SLE and correlates with disease activity
Complement C3	0.36	0.90-1.80 g/L	Low; indicative of active SLE, often associated with disease activity
Complement C4	0.06	0.10-0.40 g/L	Low; indicative of active SLE, often associated with disease activity

Therapy was initiated with a daily dose of 65 mg of methylprednisolone (1.5 mg/kg/day) and 200 mg of Plaquenil, along with monitoring of the pleura and abdomen. In case of changes in kidney function tests, azathioprine or another immunosuppressive drug would be considered for addition to the treatment regimen.

## Discussion

SLE is a complex autoimmune disease with a broad spectrum of clinical and serologic manifestations, affecting virtually every organ system. The disease is known for its unpredictable course, characterized by periods of remission and relapse, with clinical presentations ranging from mild to severe [4]. The heterogeneity of SLE can pose significant diagnostic challenges, particularly when it presents with uncommon manifestations such as PPMS.

PPMS is an exceptionally rare clinical syndrome, first described by Tjalma in 2005, characterized by the triad of pleural effusion, ascites, and elevated CA-125 levels in the absence of a benign or malignant ovarian tumor [5]. This syndrome is essentially a variant of Meigs’ syndrome, distinguished by the lack of ovarian pathology and the underlying association with SLE. The pathophysiology of PPMS in SLE remains incompletely understood, but it is hypothesized that the elevated CA-125 levels are due to the activation of mesothelial cells, which line the peritoneal, pleural, and pericardial cavities. Cytokines such as interleukin-1 (IL-1) and interferon-gamma (IFN-γ) have been implicated in increasing CA-125 expression in human peritoneal mesothelial cells, leading to the clinical manifestations observed in PPMS [6,7].

In clinical practice, the presence of ascites and pleural effusion, coupled with elevated CA-125 levels, often raises concerns about gynecological malignancies, particularly ovarian cancer [8]. However, in the context of SLE, it is crucial to consider PPMS as a differential diagnosis to prevent misdiagnosis and the subsequent initiation of inappropriate interventions. The differential diagnosis should also include other SLE-related complications, such as lupus nephritis, which can present with nephrotic syndrome, protein-losing enteropathy (PLE), and lupus peritonitis. These conditions can similarly lead to fluid accumulation in the body cavities and must be carefully distinguished from PPMS through appropriate diagnostic testing [9]. Additionally, pancreatitis should be considered in the differential diagnosis due to the patient’s gastrointestinal symptoms, as it has been reported in patients with SLE [10].

In the present case, the patient’s presentation with pleural effusion, significant ascites, and markedly elevated CA-125 levels, alongside the absence of gynecological malignancy, pointed toward a diagnosis of PPMS. This diagnosis was further supported by the serological evidence of SLE, including a positive ANA test, elevated anti-dsDNA levels, and hypocomplementemia, which collectively met the ACR/EULAR classification criteria for SLE [11].

The management of PPMS is largely focused on controlling the underlying SLE. In this case, the patient was started on high-dose corticosteroids (methylprednisolone) and an antimalarial (Plaquenil), both of which are standard therapies for managing SLE and its complications [12]. The use of immunosuppressive agents such as azathioprine may be considered in the future if there are significant changes in the patient’s kidney function or if the disease does not adequately respond to initial therapy [13].

This case underscores the importance of considering PPMS in the differential diagnosis of patients with unexplained pleural effusion and ascites, particularly in those with known or suspected SLE. Increased awareness and recognition of this rare syndrome can lead to more accurate diagnoses, appropriate treatment, and the avoidance of unnecessary invasive procedures. Furthermore, it highlights the need for continued research into the pathophysiology of PPMS to better understand its etiology and improve patient outcomes [14].

## Conclusions

PPMS is a very rare diagnosis, with only a few cases described in the literature. It is an important differential diagnosis in patients with pleural effusion, ascites, and elevated CA-125 levels, particularly in the context of SLE. This case highlights the need for careful consideration of PPMS to avoid misdiagnosis and unnecessary invasive procedures. The patient’s diverse symptoms and the eventual diagnosis of SLE emphasize the complexity of this condition. Increased awareness of PPMS can facilitate more accurate diagnoses and better clinical decision-making.
